# Characteristics of diffuse hemispheric gliomas, H3 G34-mutant in adults

**DOI:** 10.1093/noajnl/vdab061

**Published:** 2021-04-19

**Authors:** Thiébaud Picart, Marc Barritault, Delphine Poncet, Lise-Prune Berner, Cristina Izquierdo, Emeline Tabouret, Dominique Figarella-Branger, Ahmed Idbaïh, Franck Bielle, Véronique Bourg, Fanny Burel Vandenbos, Elizabeth Cohen-Jonathan Moyal, Emmanelle Uro-Coste, Jacques Guyotat, Jérôme Honnorat, Mathieu Gabut, David Meyronet, François Ducray

**Affiliations:** 1 Department of Neurosurgery, Hôpital Neurologique Pierre Wertheimer, Hospices Civils de Lyon, Bron, France; 2 Cancer Initiation and Tumoral Cell Identity Department, Cancer Research Centre of Lyon (CRCL) INSERM 1052, CNRS 5286, Lyon, France; 3 University Claude Bernard Lyon I, Villeurbanne, France; 4 Department of Molecular Biology, Groupe Hospitalier Est, Hospices Civils de Lyon, Bron, France; 5 INSERM 1052, CNRS 5286, Signaling, metabolism and tumor progression Centre Léon Bérard, Centre de Recherche en Cancérologie de Lyon, Lyon Cedex 08, France; 6 Department of Neuroradiology, Hôpital Neurologique Pierre Wertheimer, Hospices Civils de Lyon, Bron, France; 7 Department of Neurooncology, Hôpital Neurologique Pierre Wertheimer, Hospices Civils de Lyon, Bron, France; 8 Department of Neuroscience Hospital Universitari Germans Trias i Pujol, Universitat Autònoma de Barcelona, BarcelonaSpain; 9 Department of Neurooncology, AP-HM, Hôpital de la Timone, Marseille, France; 10 Aix-Marseille University, CNRS UMR 7051, Institut de Neurophysiopathologie, Marseille, France; 11 Aix-Marseille Univ, APHM, CNRS, INP, Inst Neurophysiopathol, CHU Timone, Service d’Anatomie Pathologique et de Neuropathologie, Marseille, France; 12 Sorbonne Université, Inserm, CNRS, UMR S 1127, Institut du Cerveau et de la Moelle épinière, ICM, AP-HP, Hôpitaux Universitaires La Pitié Salpêtrière - Charles Foix, Service de Neurologie 2-Mazarin, Paris, France; 13 Department of Neuropathology, AP-HP, Hôpitaux Universitaires La Pitié Salpêtrière - Charles Foix, Paris, France; 14 Sorbonne University, Inserm U1127, CNRS, UMR 7225, Université Paris 06 4 Place Jussieu, Paris, France; 15 Department of Neurology, Hôpital Pasteur, Nice, France; 16 Department of Neuropathology, Hôpital Pasteur, Nice, France; 17 Université Côte D’Azur, CNRS, INSERM, Institut de Biologie Valrose, Nice, France; 18 Department of Radiation Oncology, Institut Claudius Regaud/Institut Universitaire du Cancer de Toulouse – Oncopôle, Toulouse, France; 19 Centre de Recherches contre le Cancer de Toulouse, INSERM U1037, Toulouse, France; 20 Department of Pathology, CHU Toulouse, Institut Universitaire du Cancer-Oncopole, Toulouse, France; 21 Institut NeuroMyoGène – Equipe Synaptopathies et autoanticorps, INSERM U1217 / UMR CNRS 5310, Lyon, France; 22 Department of Pathology and Neuropathology, Groupe Hospitalier Est, Hospices Civils de Lyon, Bron, France

**Keywords:** diffuse hemispheric glioma, H3.3 G34R/V mutation, H3.3 K27M mutation, PNET, survival

## Abstract

**Background:**

Diffuse hemispheric gliomas, H3 G34-mutant (DHG H3G34-mutant) constitute a distinct type of aggressive brain tumors. Although initially described in children, they can also affect adults. The aims of this study were to describe the characteristics of DHG H3G34-mutant in adults and to compare them to those of established types of adult WHO grade IV gliomas.

**Methods:**

The characteristics of 17 adult DHG H3G34-mutant, 32 H3.3 K27M-mutant diffuse midline gliomas (DMG), 100 IDH-wildtype, and 36 IDH-mutant glioblastomas were retrospectively analyzed.

**Results:**

Median age at diagnosis in adult DHG H3G34-mutant was 25 years (range: 19–33). All tumors were hemispheric. For 9 patients (56%), absent or faint contrast enhancement initially suggested another diagnosis than a high-grade glioma, and diffusion-weighted imaging seemed retrospectively more helpful to suspect an aggressive tumor than MR-spectroscopy and perfusion MRI. All cases were IDH-wildtype. Most cases were immunonegative for ATRX (93%) and Olig2 (100%) and exhibited *MGMT* promoter methylation (82%). The clinical and radiological presentations of adult DHG H3G34-mutant were different from those of established types of adult grade IV gliomas. Median overall survival of adult DHG H3G34-mutant was 12.4 months compared to 19.6 months (*P* = .56), 11.7 months (*P* = .45), and 50.5 months (*P* = .006) in H3.3 K27M-mutant DMG, IDH-wildtype, and IDH-mutant glioblastomas, respectively.

**Conclusions:**

Adult DHG H3G34-mutant are associated with distinct characteristics compared to those of established types of adult WHO grade IV gliomas. This study supports considering these tumors as a new type of WHO grade IV glioma in future classifications.

**Key Points**

- Adult diffuse gliomas G34-mutant display a misleading radiological presentation.
- Adult diffuse gliomas G34-mutant differ from other grade IV gliomas.
- Adult diffuse gliomas G34-mutant have a poor outcome.

**Importance of the Study**

In future classifications, diffuse gliomas H3 G34-mutant will be considered as a new WHO grade IV tumor type. Since the median age at diagnosis in diffuse hemispheric gliomas, H3 G34-mutant is around 20 years, half of the patients are followed by adult neuro-oncologists. This study provides a detailed analysis of clinical, radiological, and survival of a series of 17 adult diffuse hemispheric gliomas, H3 G34-mutant. The results highlight that adult diffuse hemispheric gliomas, H3 G34-mutant frequently display a misleading radiological presentation that can lead to a potentially detrimental diagnostic delay. Although these gliomas have a poor prognosis, similar to that of H3.3 K27M-mutant diffuse midline gliomas and IDH-wildtype glioblastomas, they display distinct characteristics compared to established types of WHO grade IV adult gliomas.

Recurrent somatic gain-of-function mutations in the genes encoding the histone H3 variants H3.3 and H3.1 are encountered in and characterize 2 different types of high-grade gliomas that have initially been described in the pediatric population but can also affect adults.^1–4^ Lysine to methionine substitution at codon 27 in the *H3F3A* or in the *HIST1H3B/C* genes (H3.3 K27M mutation) are associated with aggressive diffuse midline gliomas and define “Diffuse midline glioma, H3.3 K27M-mutant” which corresponds to grade IV in the revised 2016 World Health Organization (WHO) classification.^5^ In contrast, glycine to arginine or valine substitutions at codon 34 in the *H3F3A* gene (H3.3 G34 mutation) are associated with non-midline hemispheric high-grade gliomas.^6–8^ H3.3 K27M and H3.3 G34 mutations are mutually exclusive and exclusive with IDH mutations.^1,9,10^ H3.3 G34 mutations are about twice less frequent than H3.3 K27M mutations. They are encountered in less than 1% of all gliomas but in 15% of high-grade gliomas in adolescents and young adults.^7,11,12^

In the 2016 WHO classification, H3.3 G34-mutant diffuse gliomas were not considered as a distinct tumor type and most cases are currently classified as IDH-wildtype glioblastomas.^5^ However, in future classifications, H3.3 G34-mutant diffuse gliomas will be individualized. In its sixth update, cIMPACT-NOW (the consortium to Inform Molecular and Practical Approaches to CNS Tumor Taxonomy—Not Official WHO) indeed recommended to consider H3.3 G34-mutant diffuse gliomas as a new WHO grade IV tumor type that should be referred to as “Diffuse hemispheric glioma, H3.3 G34-mutant” and the grade will be given in arabic.^13^ In the fifth edition of the CNS WHO classification, which is in progress, the suggested name is consequently “Diffuse hemispheric glioma, H3G34-mutant,” grade 4. Since the median age at diagnosis in diffuse hemispheric gliomas, H3 G34-mutant is around 18–19 years, half of the patients are followed by adult neuro-oncologists.^3,4,6,10^ Yet, the characteristics and the outcome of adult diffuse hemispheric gliomas, H3 G34-mutant remain to be fully described.^14–16^ Consequently, the aim of this study was to report the characteristics of diffuse hemispheric gliomas, H3 G34-mutant in adults and compare them to those of established types of adult WHO grade IV gliomas.

## Material and Methods

### Patient Identification

Adult patients (≥18-year-old) diagnosed with diffuse hemispheric glioma, H3 G34-mutant were retrospectively identified in 5 French University Hospitals (Lyon, Marseille, Nice, Paris, and Toulouse) between January 2012 and May 2018. Their clinical, radiological, histological, and molecular characteristics were retrospectively reviewed.

The characteristics of adult diffuse hemispheric gliomas, H3 G34-mutant were compared to those of retrospective contemporaneous series of H3.3 K27M-mutant diffuse midline gliomas (DMG), IDH-wildtype, and IDH-mutant glioblastomas diagnosed in Lyon University Hospital according to the revised WHO 2016 classification. The inclusion criteria were the following: (1) age ≥ 18 years, (2) available MRI for radiological review, (3) and available *TERT* promoter mutation status for IDH-wildtype glioblastomas.

Brain MRI scans were independently reviewed by 3 investigators (T.P., C.I., and L.P.B.) for the location of T2/fluid-attenuated inversion recovery (FLAIR) hyperintensity, the presence and type (fainted, nodular, or ring-like) of parenchymal contrast enhancement, the presence of apparent diffusion coefficient (ADC) restriction, as well as abnormalities on proton magnetic resonance spectroscopy (1H-MRS) and, dynamic contrast-enhanced perfusion MRI. For patients with available MRI follow-up, tumor progression was defined according to RANO criteria.^17^

### Molecular Analysis

DNA was extracted from formalin-fixed paraffin-embedded samples using a standard protocol (QIAmp DNA mini Kit, Qiagen). After PCR amplification, the mutations of codon 132 of IDH1, codon 172 of IDH2, codons 27 and 34 of H3F3A, codon 28 of HIST1H3B, codon 600 of BRAF, as well as mutational hotspots C228 and C250 of the *TERT* promoter were investigated by single-nucleotide primer extension with the ABI PRISM SNaPshot Multiplex kit, Applied Biosystems.^18^

*MGMT* promoter methylation was assessed by pyrosequencing, which was performed using the PyroMark Q96 MGMT kit (Qiagen) on a PSQTM96 MA system (Biotage).^19^*EGFR* gene amplification was detected by using SYBR Green real-time quantitative polymerase chain reaction (qPCR) analysis (Absolute SYBR Green Rox Mix; Abgene) with the following set of primers EGFR-F: GTGCAGATCGCAAAGGTAATCAG, EGFR-R: GCAGACCGCATGTGAGGAT; hydrolysis probe: FAM CCCCTCCCCCGTATCTC.^20^

### Statistical Analysis

Categorical comparisons were performed using the Fisher’s exact test or the Chi2 test when the conditions of application were met. The nonparametric Wilcoxon test was used for quantitative variable as the hypothesis of normality of distribution was not verified. Overall survival and progression-free survival were represented with Kaplan–Meier plots and compared using the log-rank test.

The statistical tests were bilateral and the level of significance was set at 5% (*P* < .05). Statistical analyses were conducted using SAS version 9.4 (SAS Institute Inc.).

### Standard Protocol Approvals and Registrations

Study design and manuscript organization were guided by the STROBE statement on cohort studies. The study was approved by the Institutional Review Board (local number 19–113). Informed consents were obtained from alive patients.

## Results

### Patients and Tumor Characteristics

A total of 17 adult patients diagnosed with a diffuse hemispheric glioma, H3 G34-mutant were retrospectively identified. Their characteristics are summarized in Table 1. Median age at diagnosis was 25 years (range: 19–33). Most patients were aged ≤ 30 years (*n* = 13, 76%). Clinical presentation consisted of epileptic seizure (*n* = 9, 53%), focal deficit (*n* = 5, 29%), and acute intracranial hypertension (*n* = 3, 18%) that was imputable to a massive tumor hemorrhage in 2 patients (12%).

**Table 1.** Characteristics of the 17 Included Adult Patients Diagnosed With Diffuse Hemispheric Glioma, H3 G34-mutant

Patients	Sex	Age (years)	Suspected diganosis	Tumor location	Gadolinium menhancement	Presence of a cyst	Presence of necrotic areas	Restriction of diffusion	Surgical management	Microscopic aspect	Ki67 index	Olig2 staining	TP53 staining	IDH mutation	ATRX loss	EGFR amplification	MGMT methylation	Progression free survival (months)	Overall survival (months)
1	M	29	HGG	PO	Yes	Yes	No	-	TR	Monstro	35%	0%	80%	No	Yes	No	Yes	97	99^c^
2	F	25	HGG	FP	Yes	No	No	-	TR	PNET	60%	0%	80%	No	Yes	No	Yes	40.8	53.4
3	F	23	MS	FP	No	No	No	Yes(F)	B	PNET	40%	0%	60%	No	-	No	Yes	9.3	11.1
4	M	33	LE	TI	No	No	No	Yes	PR	PNET	75%	0%	100%	No	Yes	No	Yes	4.5	11.9
5	F	29	LGG/MS	FP^a^	No	No	No	Yes	B	PNET	20%	0%	100%	No	Yes	No	-	5.7	50.4
6	F	30	HGG	FC^b^	Yes	Yes	Yes	Yes(F)	B	Undiff	28%	0%*	80%	No	No	No	Yes	9.5	27.5
7	M	22	H/HGG	TI	Yes(F)	No	No	Yes	TR	PNET	40%	0%	100%	No	Yes	Yes	Yes	23.1	28.8
8	M	33	PCNSL	FC^b^	Yes(F)	No	No	Yes(F)	B	Oligoid	60%	0%*	100%	No	Yes	No	Yes	4.9	6.3
9	M	21	H/AVM	FP	-	No	No	-	TR	Monstro	95%	0%	100%	No	Yes	No	Yes	24	30^c^
10	M	19	LGG	FP^b^	No	No	No	Yes(F)	B	PNET	40%	0%	100%	No	Yes	Yes	No	7.1	9.0
11	F	37	LGG	FP	No	No	No	No	B	A-III	30%	0%*	10%	No	Yes	No	Yes	26.2	55.4
12	M	31	HGG	TI^a^	Yes(F)	No	No	Yes(F)	B	Undiff	40%	0%	90%	No	-	No	No	8.7	12.4
13	M	29	A	FP^a^	Yes(F)	No	No	Yes(F)	B	PNET	50%	0%	90%	No	Yes	-	-	8.8	12.5
14	M	18	MD	PC^b^	Yes(F)	No	No	Yes(F)	B	Undiff	25%	0%	40%	No	Yes	-	-	3.2	4.2
15	M	22	H/HGG	FP	Yes	No	No	Yes	B	PNET	25%	0%	5%	No	Yes	-	-	0.1	0.1
16	F	19	PCNSL	FP	Yes(F)	No	No	Yes(F)	B	Monstro	15%	0%	90%	No	Yes	No	-	6.4	8.4
17	M	18	-	-	-	-	-	-	B	Undiff	-	-	-	No	-	No	-	11.4	14.4

Sex. F: Female, M: Male;

Initially suspected diagnosis. A: Abscess, AVM: Arterio-venous Malformation, H: Hematoma, HGG: High-grade glioma, LE: Limbic encephalitis, LGG: Low-grade glioma, MD: Metabolic disease, MS: Multiple sclerosis, PCNSL: Primary central nervous system lymphoma;

Tumor location. FC: fronto-callosal, FP: fronto-parietal, PO: parieto-occipital, TI: temporo-insular, ^a^ indicates tumors that were only cortical at diagnosis, ^b^ indicates midline invasion;

Gadolinium enhancement. (F): Faint;

Restriction of Diffusion. (F): Focal;

Surgical management. B: Biopsy, PR: Partial Resection, TR: Total Resection;

Microscopic aspect. A-III: Grade III Astrocytoma, Monstro: Monstroscellular glioblastoma, Oligoid: Glioblastoma with oligoid morphology, PNET: Glioblastoma with Primitive Neuro-Ectodermic Tumor-like foci, Undiff: Glioblastoma with undifferenciated morphology;

Olig2 staining. * indicates cases containing Olig2-imunopositive reactive glial cells

Overall survival. ^c^ indicates patients who are still alive.

**Figure 1.**
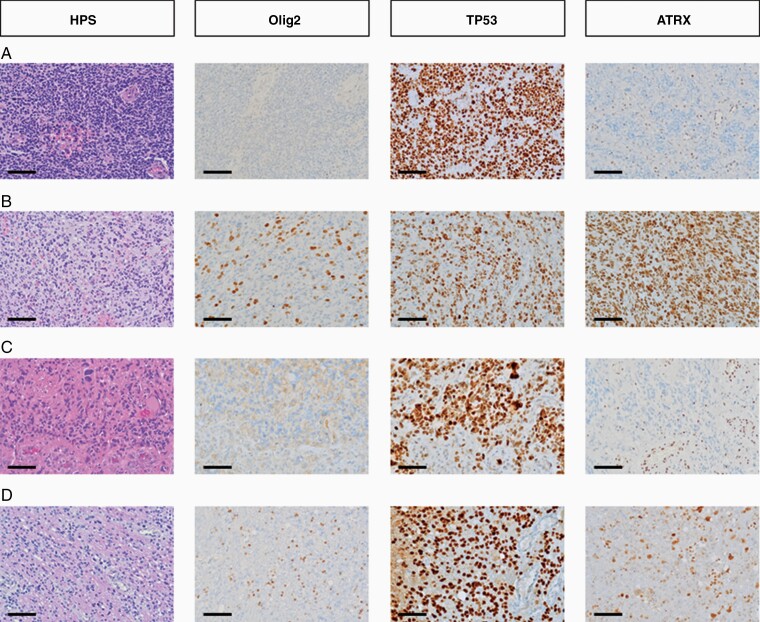
Histological characteristics of adult diffuse hemispheric gliomas, H3 G34-mutant (Scale bar = 100 µm). (A) PNET-like morphology, characterized by small round cells, with anaplastic features including endothelial cell proliferation, negative Olig2 immunostaining, positive TP53 staining and ATRX loss. (B) Undifferenciated glial morphology, characterized by irregularly shaped nuclei and poorly delimited cytoplasms with anaplastic features including endothelial cell proliferation, positive Olig2 (reactive glial cells), ATRX, and TP53 stainings. (C) Monstrocellular morphology, characterized by multinucleated cells, with anaplastic features including endothelial cell proliferation, necrosis, negative Olig2 immunostaining, positive TP53 staining, and ATRX loss. (D) Oligoid morphology, characterized by small cells with a clear peri-nuclear halo, with anaplastic features including endothelial cell proliferation and 2 mitoses, positive Olig2 (reactive glial cells) and TP53 immunostaining and ATRX loss.

Histologically, all cases presented as a diffusely infiltrating high-grade glioma. Mitotic activity was observed in all cases, and microvascular proliferation and/or necrosis in all but one case (94%). All cases were IDH-wildtype (17/17). According to the 2016 WHO classification, one case was diagnosed as an IDH-wildtype anaplastic astrocytoma and 16 as IDH-wildtype glioblastomas. Among the latter cases, 8 tumors displayed a PNET-like morphology (50%), 4 displayed an undifferentiated morphology (25%), 3 contained multinucleated giant cells (19%), and 1 displayed an oligodendroglial appearance (6%). These features are illustrated in Figure 1. Median Ki67 labeling was 42% (range: 15–95%). Olig2 immunostaining was completely negative in 13/16 cases (81%) and focally positive in 3/16 cases (19%). In these cases, however, Olig2 positivity corresponded to reactive glial cells and after review they were finally considered as negative. TP53 staining was positive (>10%) in 14/16 cases (88%) and ATRX expression was lost in 13/14 cases (93%). Finally, all cases displayed ATRX loss and/or TP53-positive immunostaining. The *H3F3A* mutation was a G34R mutation in all cases and was conserved at recurrence in the 2 patients from the series who underwent a second surgery. All studied cases were BRAF wildtype (*n* = 15/15) and lacked TERT promoter mutations (*n* = 10/10). An *EGFR* amplification was present in 2/14 patients (14%) and *MGMT* promoter was methylated in 9/11 patients (82%).

### Radiological Presentation

Initial MRI scans were available for review for 16/17 patients. Radiological characteristics are summarized in Table 1. One patient (patient 6) underwent a brain MRI which was normal, 3 years before diagnosis. At diagnosis, all tumors had a hemispheric location and were monocentric. Midline involvement was observed in 4 patients but always as an extension of a primarily hemispheric tumor. Most cases demonstrated no or only a faint contrast-enhancement (*n* = 11/15, 73%) and presented as cortico-subcortical (*n* = 13/16, 81%), poorly delimited (*n* = 9/16, 69%), infiltrative lesions that were mostly located in the fronto-parietal (*n* = 11/16, 69%) or in the temporo-insular lobes (*n* = 3/16, 19%). All but one tumor (*n* = 12/13, 92%) harbored areas of ADC restriction on diffusion-weighted imaging. Only half of the cases in which perfusion MRI and/or MR spectroscopy were performed presented an elevated (>1.7) intratumoral relative cerebral blood flow (50%, *n* = 4/8) or a metabolic profile suggestive of an aggressive tumor (elevated Choline/N-Acetyl-Aspartate ratio > 2 and/or of lipid/lactate peaks, 44%, *n* = 4/9).

**Figure 2.**
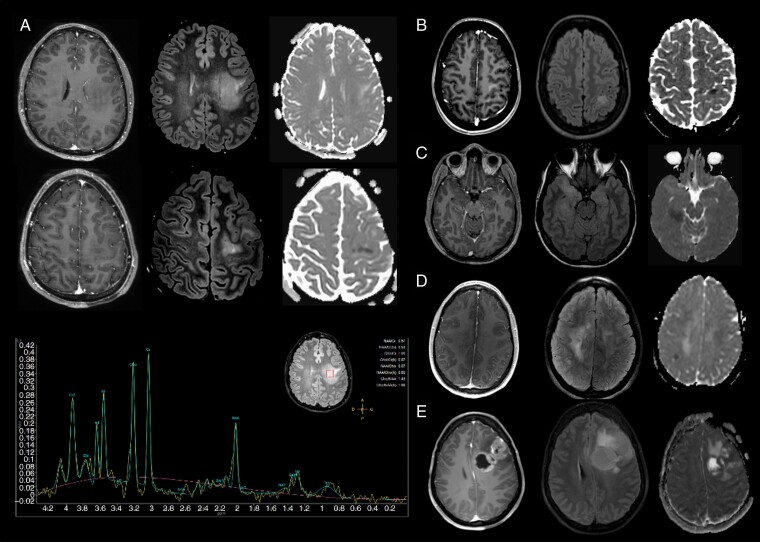
Radiological characteristics of adult diffuse hemispheric gliomas, H3 G34-mutant For each case, T1-weighted contrast-enhanced MRI (left), T2-weighted FLAIR MRI (middle) and ADC map (right) are shown. (A) Frontal cortico-subcortical, poorly delimited, diffuse hemispheric glioma, H3 G34-mutant without contrast enhancement, displaying focal ADC restriction and a metabolic profile on MR spectroscopy demonstrating no Choline/Creatinine ratio increase but an elevated Choline/N-Acetyl-Aspartate ratio (2.35) and a lipid peak (patient 10). In this patient, a low-grade glioma was initially suspected. (B–D) Three other cases of diffuse hemispheric gliomas, H3 G34-mutant with a misleading presentation, including absent or faint contrast enhancement and a focal ADC restriction. These characteristics led to suspect at first diagnosis multiple sclerosis (B—Patient 5, D—Patient 3), limbic encephalitis (C—Patient 4) or low-grade glioma (D—Patient 3). (E) Fronto-callosal diffuse hemispheric glioma, H3 G34-mutant with a presentation suggestive of high-grade glioma, including necrosis area and an evident contrast enhancement (patient 6). Three years earlier, this patient underwent an MRI which was normal.

For 6 patients, the initial radiological presentation led to suspect a high-grade glioma. For 10 cases, however, the young age of the patient, absent or faint contrast enhancement (*n* = 9) or the presence of an isolated hemorrhage (*n* = 1) led to suspect another diagnosis (Figure 2). In these patients, the initially suspected diagnoses were the following: low-grade glioma (*n* = 3), primary CNS lymphoma (*n* = 2), multiple sclerosis (*n* = 2), limbic encephalitis (*n* = 1), metabolic disease (*n* = 1), and ruptured arterio-venous malformation (*n* = 1). Subsequently, in 8 patients, the decision was made to perform an MRI follow-up leading to a median delay of 2.3 months before histological diagnosis. Retrospectively, in all but one of these patients, the initial MRI demonstrated focal ADC restriction that could have led to suspect an aggressive glioma. In contrast, an elevated intra-tumoral relative cerebral blood flow and an aggressive metabolic profile on MR spectroscopy were both present in only 2/8 patients (25%).

Among the 11 patients with initially absent or faint contrast-enhancement, 8 underwent a repeated MRI before (*n* = 4) or after (*n* = 4) histological diagnosis that demonstrated the apparition of a ring-like or nodular contrast enhancement in all of the patients after a median interval of 2.6 months (range: 0.8–5.3 months).

### Treatment and Outcome

A total of 12 patients underwent a biopsy (70%), 1 underwent a partial (6%) and 4 a complete resection (24%). One patient presented with a brain hematoma leading to neurological worsening after biopsy and one patient presented a lethal refractory raised intracranial pressure after biopsy. In the 15 remaining patients, postsurgical course was uneventful. Postoperative oncological treatment consisted of temozolomide radio-chemotherapy (*n* = 13, 82%), radiotherapy alone (*n* = 1, 6%), radiotherapy followed by PCV (procarbazine, CCNU, and vincristine) chemotherapy (*n* = 1, 6%), and temozolomide chemotherapy alone (*n* = 1, 6%). No severe adverse effect was reported during the oncological treatment and no patient developed drug-resistant epilepsy.

At last news, after a median follow-up of 12.7 months, tumor progression was diagnosed in 17 patients and 15 patients had died. The median progression-free survival was 8.8 months and the median overall survival was 12.4 months, in the entire series. For the 13 patients who were treated with temozolomide radio-chemotherapy, the median overall survival was 25 months. The pattern of tumor progression was analyzed for 15 patients and consisted of gliomatosis cerebri (*n* = 9, 60%) with contralateral hemispheric (*n* = 4, 27%) or infratentorial (*n* = 5, 33%) infiltration, local recurrence (*n* = 3, 20%), distant progression (*n* = 3, 20%), and leptomeningeal dissemination (*n* = 4, 26%). At progression, treatment consisted of chemotherapy alone (*n* = 8, 53%), chemotherapy combined with cyberknife radiation (*n* = 1, 8%) or conformational radiotherapy (*n* = 1, 8%), surgery followed by chemotherapy (*n* = 2), radiotherapy at a remote site (*n* = 1, 8%), or palliative cares (*n* = 3, 23%).

### Comparison of Adult Diffuse Hemispheric Gliomas, H3 G34-mutant With Established Types of Adult WHO Grade IV Gliomas

**Figure 3.**
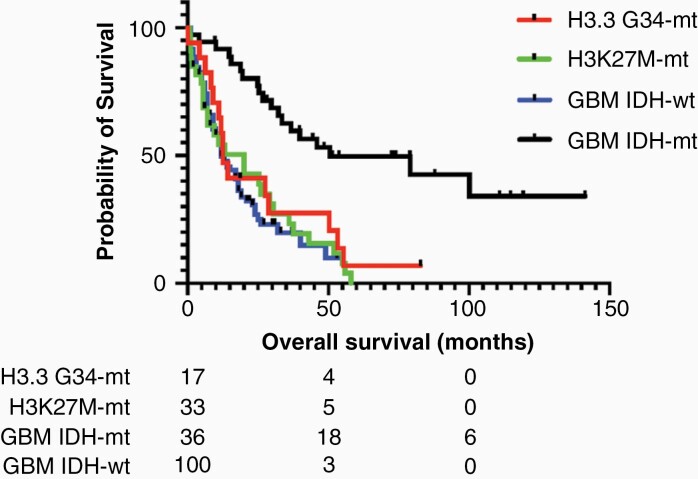
Kaplan–Meier survival analysis of diffuse hemispheric gliomas, H3 G34-mutant, H3.3 K27M-mutant diffuse midline gliomas, IDH-mutant glioblastomas, and IDH-wildtype glioblastomas patients. Patients at risk are indicated below.

As given in Table 2, several characteristics of adult diffuse hemispheric gliomas, H3 G34-mutant differed significantly from those of established types of adult WHO grade IV gliomas. Compared to H3.3 K27M-mutant DMG, IDH-mutant and IDH-wildtype glioblastomas, diffuse hemispheric gliomas, H3 G34-mutant occurred in younger patients, more frequently involved the parietal lobe, more frequently presented as a hemorrhagic tumor at diagnosis, less frequently demonstrated contrast-enhancement and necrosis, more frequently displayed ADC restriction and less frequently expressed Olig2. In addition, *TERT* promoter mutations were lacking in diffuse hemispheric gliomas in contrast to IDH-wildtype glioblastomas while *MGMT* promoter methylation was more frequent in diffuse hemispheric gliomas, H3 G34-mutant than in H3.3 K27M-mutant DMG and IDH-wildtype glioblastomas. Median overall survival of adult diffuse hemispheric gliomas, H3 G34-mutant was 12.4 months compared to 19.6 months (*P* = .56), 11.7 months (*P* = .45), and 50.5 months (*P* = .006) in H3.3 K27M-mutant DMG, IDH-wildtype glioblastomas, and IDH-mutant glioblastomas, respectively (Figure 3). The comparison yielded similar results when the analysis was restricted to H3.3 K27M-mutant DMG and IDH-mutant glioblastomas with comparable age (ie, aged < 35 years, data not shown). In IDH-wildtype glioblastomas, the number of patients aged < 35 years was too limited to allow this analysis.

**Table 2.** Comparison of the Characteristics of Adult Diffuse Hemispheric Gliomas, H3 G34-Mutant with Those of H3.3 K27-mutant DMG, IDH1/2-mutant Glioblastomas and pTERT-mutant Glioblastomas

	H3.3 G34-mutant gliomas (*n* = 17)	H3.3 K27-mutant DMG (*n* = 33)	*P* ^a^	IDH-mutant glioblastomas (*n* = 36)	*P* ^b^	IDH-wt glioblastomas (*n* = 100)	*P* ^c^
Median age at diagnosis (years)	25.8 (SD = 5.9)	34.8 (SD = 16.5)	**.005**	38.5 (SD = 13.7)	**<.001**	66.4 (SD = 11.8)	**<.001**
Sex							.30
Male	11/17 (65%)	15/33 (45%)	.24	13/36 (36%)	.09	49/100 (49%)	
Female	6/17 (35%)	18/33 (55%)		23/36 (64%)		51/100 (51%)	
Location							
Frontal	11/16 (69%)	0/33 (0%)	**<.001**	24/36 (67%)	.99	48/100 (48%)	.27
Parietal	11/16 (69%)	0/33 (0%)	**<.001**	7/36 (19%)	**.001**	22/100 (22%)	**<.001**
Temporal	3/16 (19%)	2/33 (6%)	.31	11/36 (31%)	.51	32/100 (32%)	.25
Occipital	1/16 (6%)	0/33 (0%)	.32	1/36 (3%)	.52	14/100 (14%)	.69
Corpus callosum	3/16 (19%)	0/33 (0%)	**.03**	11/36 (31%)	.50	33/100 (33%)	.38
Midline	4/16 (25%)	33/33 (100%)	**<.001**	10/36 (28%)	.99	10/100 (10%)	.11
Initial hemorrhage							**.01**
Yes	3/16 (19%)	0/33 (0%)	**.03**	2/36 (6%)	.16	2/100 (2%)	
No	13/16 (81%)	33/33 (100%)		34/36 (94%)		98/100 (98%)	
Radiological features							
CE	4/15 (27%)	18/29 (62%)	.05	27/36 (75%)	**.002**	93/100 (93%)	**<.001**
Cyst	2/16 (12%)	3/28 (11%)	.60	7/36 (19%)	.70	8/100 (8%)	.60
Necrosis	1/16 (6%)	10/28 (36%)	.03	14/36 (39%)	**.02**	70/100 (70%)	**<.001**
ADC restriction	12/13 (92%)	3/23 (13%)	**<.001**	4/30 (13%)	**<.001**	35/68 (51%)	**.006**
IHC features							
OLIG2 positivity	3/16 (19%)	29/29 (100%)	**<.001**	36/36 (100%)	**.03**	89/91 (98%)	**<.001**
Molecular characteristic							
IDH mut.	0/17 (0%)	0/33 (0%)	.99	-	-	-	-
EGFR amplif.	2/14 (14%)	0/33 (0%)	.08	2/34 (6%)	.57	34/96 (35%)	.13
pTERT mut.	0/15 (0%)	2/30 (7%)	.99	1/28 (4%)	.99	88/100 (88%)	**<.001**
MGMT meth.	9/11 (82%)	4/31 (13%)	**.001**	27/32 (84%)	.99	46/93 (49%)	.05
BrafV600E mut.	0/10 (0%)	0/30 (0%)	.99	0/18 (0%)	.99	1/59 (2%)	.99
Median Overall Survival (months)	12.4 95%CI [6.3–27.5]	19.6 95%CI [6.1–29.1]	.56	50.5 95% CI [45.9–79.2]	**.006**	11.7 95%CI [9.3–17.5]	.45

ADC, Apparent diffusion coefficient; CE, Contrast enhancement; SD, Standard deviation.

^a^H3.3 K27-mutant DMG compared to diffuse hemispheric gliomas, H3 G34-mutant, ^b^ IDH-mutant glioblastomas compared to diffuse hemispheric gliomas, H3 G34-mutant, ^c^ IDH-wt glioblastomas compared to diffuse hemispheric gliomas, H3 G34-mutant.

Significant *P*-values are indicated in bold characters.

## Discussion

Diffuse hemispheric gliomas, H3 G34-mutant are rare brain tumors that affect children and adolescents but also adults.^4,10,12,15^ In this study, that is the first dedicated to adult cases, we show that adult diffuse hemispheric gliomas, H3 G34-mutant frequently displayed a misleading radiological presentation, had distinct characteristics compared to established types of WHO grade IV adult gliomas and a poor prognosis.

### Radiological Presentation

The radiological characteristics of adult diffuse hemispheric gliomas, H3 G34-mutant in the present series are consistent with those reported in previous studies, although these studies were not dedicated to adults (Table 3). In the present series, adult diffuse hemispheric gliomas, H3 G34-mutant were constantly located within cerebral hemispheres,^3,4,6,8–10,14^ frequently affected the parietal and temporal lobes,^4,6–8,11,15,21,22^ frequently demonstrated no or faint contrast-enhancement,^7,15^ not rarely presented a massive initial hemorrhage^23^ and, frequently presented an unusual presentation for a high-grade glioma.^7,15,23^ Actually, less than half of cases fulfilled the imaging criteria of high-grade glioma which led in some cases to a potentially detrimental diagnostic delay. Patients for whom the radiological presentation was misleading typically presented with an MRI aspect that has been previously described as “gliomatosis cerebri-like,” consisting of a non-contrast enhancing ill-defined infiltrating lesion that led to suspect another diagnosis than a high-grade glioma because of the young age of the patients.^15^ Nontumoral diagnoses, including inflammatory, infectious, vascular and metabolic diseases, could be initially suspected. Retrospectively, a striking feature in these patients that could have raised suspicions toward an aggressive hypercellular tumor^15,24^ was the presence of areas of profound focal ADC restriction. In addition to diffusion-weighted-imaging, dynamic 18F-Fluoro-Ethyl-L-Tyrosine positron emission tomography (FET-PET) may be useful to preoperatively identify non-contrast enhancing diffuse hemispheric gliomas, H3 G34-mutant as aggressive gliomas.^23^

**Table 3.** Summary of H3 G34-mutant gliomas characteristics in the present and in previously reported series*

	Present series	Chen C et al.^28^	Roux et al.^12^	Mackay et al.^22^	Vettermann et al.^23^	Mackay et al.^21^	Puntonet et al.^15^	Neumann et al.^11^	Korshunov et al.^6^	Sturm, Schwartzentruber et al.^4^	Summary
		2020	2020	2018	2018	2017	2017	2016	2016	2012	
Population	Adult	Pediatric, adult	15–25 years	Pediatric	Pediatric, adult	Pediatric, adult	Pediatric, adult	<30 years	Pediatric, adult	Pediatric, adult	
*N*	17	95	11	7	8	67	12	12	81	18	
Median age (years, range)	25 (19–33)	-	17.6	12	27 (9–52)	15 (7–31)	16 (6–31)	14.5	19 (9–51)	18 (9–42)	12–27
Radiological characteristics											
Main location	FP	-	FP	TP	TP (50%)	TP	T	TP	TP (80%)	TP	FTP
Contrast-enhancement (%)	27%	-	100%	-	62%	-	37%	-	-	-	27%–100%
ADC restriction (%)	92%	-	-	-	-	-	100%	-	-	-	92%–100%
Histological and molecular characteristics (%)											
GBM-like	53%	-	72%	57%	-	-	83%	0%	50%	-	50%–83%^$$^
PNET-like	47%	-	-	-	-	-	17%	100%	50%	-	17%–50%^$$^
Olig2 immunonegative	100%	-	-	100%	-	-	100%	-	100%	100%	100%
ATRX loss or mutant	93%	84%	-	86%	-	89%	75%	91%	95%	100%	75%–100%
TERTp mutation	0%	-	-	-	-	-	-	-	-	-	-
TP53 expression or mutant	88%	95%	-	-	-	89%	-	54%	88%	100%	54%–100%
EGFR amplification	14%	-	-	-	-	3%	-	-	4%	5%	3%–14%
PDGFRA alteration	-	44%/81%**	-	71%***		13%^$^	-	-	27%^$^	10%^$^	10%–71%
MGMT methylated	82%	-	-	50%		65%	-	-	74%	-	50%–82%
Median survival (months)	12.5	-	36.2	12	-	18	-	-	22	24	12–36.2

FP, fronto-parietal; TP, temporo-parietal; T, temporal.

*Only series with > 4 cases were included.

** PDGFRA mutations diagnosis/recurrence, *** PDGFRA mutations and amplifications, $: PDGFRA amplification, $$: excluding Neumann et al. study^11^ that focused on cases with a PNET-like presentation.

### Histo-molecular Presentation

At the histo-molecular level, it was evidenced herein that adult diffuse hemispheric gliomas, H3 G34-mutant could present a PNET-like or a GBM-like morphology, lacked Olig2 expression, were most frequently immunonegative for ATRX, immunopositive for TP53, frequently demonstrated a methylated *MGMT* promoter, and lacked *TERT* promoter mutations. These findings are consistent with those reported in previous series that included both pediatric and adult cases (Table 3).^3,4,6,7,11,15,21,22,25,26^ The absence of Olig2 expression is a hallmark of diffuse hemispheric gliomas, H3 G34-mutant. Yet, as observed in our series, reactive glial cells may wrongly suggest positivity in some cases. As highlighted in children,^27^ due to a frequent PNET-like morphology, adult diffuse hemispheric gliomas, H3 G34-mutant could have previously been misdiagnosed as CNS PNET, a now obsolete entity. Actually, in a young adult presenting with an IDH-wildtype hemispheric PNET-like tumor, the most likely diagnosis is a diffuse hemispheric glioma, H3 G34-mutant since other entities that can present as PNET-like tumors occur in younger patients.^28^ Importantly, in the present series, despite the young age of the patients and a high rate of *MGMT* promoter methylation, adult diffuse hemispheric gliomas, H3 G34-mutant had a poor prognosis. Despite many similarities, it has been shown that pediatric and adult H3.3 K27M-mutant DMG present significant differences.^29^ Compared to pediatric cases, adult H3.3 K27M-mutant DMG more frequently affect the thalamus and less frequently the pons, almost exclusively harbour *H3F3A* mutations and have a better prognosis.^29^ It remains to be determined whether this is also the case in diffuse hemispheric gliomas, H3 G34-mutant, however, these gliomas seem to be a more homogeneous entity than H3.3 K27M-mutant DMG. Age at diagnosis is narrower in H3 G34-mutant gliomas than in H3.3 K27M-mutant DMG and in a previous study neither histological appearance nor molecular characteristics were associated with age.^6^ Also consistent with this hypothesis, as detailed in Table 3, the characteristics of adult diffuse hemispheric gliomas, H3 G34-mutant in the series herein seemed very similar to those reported in previous studies that included both adult and pediatric cases^3,4,6,21,22,27^ or only pediatric cases.^3,4,6,21,22,27^ However, analysis of larger series will be necessary to assess whether there are age-related differences in H3 G34-mutant gliomas.

### Outcomes and Therapeutic Opportunities

The median overall survival of adult diffuse hemispheric gliomas, H3 G34-mutant herein is consistent with the median overall survival ranging from 12 to 36 months reported in previous series (Table 3).^3,4,6,12,21,22,26^ Although several studies suggested that diffuse hemispheric gliomas, H3 G34-mutant had a better prognosis than H3.3 K27M-mutant DMG^4,10,30,31^ the prognosis of these two tumor types was similar in the present series and was also similar to that of IDH-wildtype glioblastomas. However, the clinical, radiological and histo-molecular characteristics of diffuse hemispheric gliomas, H3 G34-mutant were very different from those of H3.3 K27M-mutant DMG, IDH-mutant, and IDH-wildtype glioblastomas. These findings support that diffuse hemispheric gliomas, H3 G34-mutant should be considered as a new WHO grade IV tumor type in future classifications.^13^

Beside gliomas, H3.3 G34 mutations are found in the majority of giant cell tumors of the bone (92%) and some osteosarcomas (5%).^32,33^ Although the oncogenesis of diffuse hemispheric gliomas, H3 G34-mutant remains to be fully understood, some therapeutic opportunities have already been identified. Histones pack DNA and are involved in transcriptional regulation and telomeres stability^10,34^ through post-translational modifications.^1,2,14,34–36^ Mutations of some histone residues, such as the H3.3 K27 and K36 residue, generate conformational changes or steric inhibition that prevent post-translational modifications and trigger deep epigenetic reprogramming.^1–3,14,34^ In contrast to the H3.3 K27 residue, the H3.3 G34 residue does not undergo post-translational modifications. However, the H3.3 G34 mutations alter accessibility of H3 K36 to histone methyltransferases affecting H3 K36 methylation and subsequently gene expression.^37^ H3.3 G34 mutations have been shown to result in the upregulation of transcription factor MYCN^38,39^ and to impair H3 K36 methylation by SETD2 which promotes aberrant PRC2 activity and drives tumor progression.^40^ H3.3 G34 mutations also promote genomic instability by blocking the interaction between H3 K36 and the mismatch repair protein MutSα.^41^ In addition, it has been shown that diffuse hemispheric gliomas, H3 G34-mutant are characterized by a global DNA hypomethylation and ATRX/DAXX mutations, resulting in alternative lengthening of telomeres (ALT).^3,4,8,25,30^ Recently, it has been shown that although considered gliomas, H3 G34-mutant tumors arise in specific interneuron progenitors, in which G34 mutations prevent neuronal differentiation and that PDGFRA signalling plays a major role in these tumors.^26^ PDGFRA activating mutations (mostly in the extracellular domain) which are present in 40% of the cases at diagnosis and, in 80% of the cases at recurrence, promote astroglial features and are potent oncogenic drivers.^26^ In the future, targeting MYCN,^38^ DNA hypomethylation,^4,8^ ALT,^3,25,30^ and PDGFRA signaling^26^ could constitute new treatment strategies in diffuse hemispheric gliomas, H3 G34-mutant. Targeting MYCN upregulation could be achieved by inhibiting kinases such as CHK1 and AURKA which stabilize MYCN^38^ or by using BRD3 and BRD4 inhibitors which play essential roles in the transcription of MYCN.^42,43^ Regarding PDGFRA signaling inhibition, given the high rate of extracellular mutations, it remains to be determined whether it could be best achieved through PDGFRA inhibitors or through inhibitors targeting downstream MAPK activation.^26^ In H3.3 K27M-mutant DMG histone deacetylase inhibitors and the selective antagonist of dopamine receptor DRD2/3 ONC201 are promising therapeutic strategies that are currently being evaluated in clinical trials.^44,45^ Whether these strategies may benefit patients with H3 G34-mutant gliomas remains to be determined.

### Study Limitations

Other than the limited sample size and its retrospective design, this study is limited by the absence of a comprehensive molecular analysis, the absence of multimodal imaging in all of the patients, and the absence of comparative pediatric series. In addition, the results will need to be validated in an independent and larger series. Nevertheless, the study provides for the first time a picture of adult diffuse hemispheric gliomas, H3 G34-mutant characteristics.

## Conclusions

Adult diffuse hemispheric gliomas, H3 G34-mutant are associated with distinct characteristics compared to those of established types of adult WHO grade IV gliomas. This study supports considering these tumors as a new type of WHO grade IV glioma in future classifications. If further studies confirm that adult and pediatric diffuse hemispheric gliomas, H3 G34-mutant share similar characteristics, it may be reasonable to include patients in the same clinical trials, irrespective of age.

## Notes

**Authorship Statement.** T.P., D.M., and F.D. designed the study. T.P., M.B., D.P., C.I., E.T., V.B., F.B.V., E.C.J.M., and J.G. substantially contributed to data acquisition. T.P., M.B., L.P.B., C.I., D.F.B., A.I., F.B., E.U.C., J.G., M.G., D.M., and F.D. interpreted the data. T.P. and F.D. drafted the manuscript. All authors revised the manuscript critically for important intellectual content and approved the final version to be published.
